# Gut Microbiome Composition in Non-human Primates Consuming a Western or Mediterranean Diet

**DOI:** 10.3389/fnut.2018.00028

**Published:** 2018-04-25

**Authors:** Ravinder Nagpal, Carol A. Shively, Susan A. Appt, Thomas C. Register, Kristofer T. Michalson, Mara Z. Vitolins, Hariom Yadav

**Affiliations:** ^1^Department of Internal Medicine-Molecular Medicine and Department of Microbiology and Immunology, Wake Forest School of Medicine, Winston-Salem, NC, United States; ^2^Department of Epidemiology and Prevention, Wake Forest School of Medicine, Winston-Salem, NC, United States; ^3^Department of Epidemiology and Prevention, Wake Forest School of Medicine, Winston-Salem, NC, United States

**Keywords:** diet, microbiome, mediterranean, primates, cynomolgus macaque, western, metabolism, microbiota

## Abstract

The mammalian gastrointestinal tract harbors a highly diverse and dynamic community of bacteria. The array of this gut bacterial community, which functions collectively as a fully unified organ in the host metabolism, varies greatly among different host species and can be shaped by long-term nutritional interventions. Non-human primates, our close phylogenetic relatives and ancestors, provide an excellent model for studying diet-microbiome interaction; however, compared to clinical and rodent studies, research targeting primate gut microbiome has been limited. Herein, we analyze the gut microbiome composition in female cynomolgus macaques (*Macaca fascicularis*; *n* = 20) after the long-term (2.5 years) consumption of diets designed to mimic recent human Western- (WD; *n* = 10) or Mediterranean-type (MD; *n* = 10) diets. Microbiome diversity in MD consumers was significantly higher by the Shannon diversity index compared to the WD consumers, with similar but non-significant trends noted for the diversity metrics of species richness (Chao 1), observed operational taxonomic units (OTUs) and phylogenetic diversity (PD) whole Tree. Compared to the MD, the WD group demonstrated a higher Firmicutes-Bacteroides ratio and a significantly higher abundance of families *Clostridiacea* and *Lactobacillaceae*. Further analyses reveal significantly higher abundance of genera *Lactobacillus, Clostridium, Faecalibacterium*, and *Oscillospira* and lower abundance of *Ruminococcus* and *Coprococcus* in MD consumers relative to WD consumers. OTUs belonging to several species also show significant differences between the two groups, with *Lactobacillus* species demonstrating a prominently higher abundance in the MD consumers. The data reveal several differences in the gut microbiome of primates consuming the two different diets and should be useful for further studies aimed at understanding the diet-microbiome-health interactions in primates.

## Introduction

The past decade has been remarkable in revealing the fundamental role of the gut microbiome (a highly complex and diverse community of microbes living within gastrointestinal tract) in human health and diseases [1, 2]. Among various intrinsic and extrinsic factors that affect gut microbiome composition and diversity, diet has received appreciable attention because of its potential influence on host health and metabolism [3, 4]. Diet shapes the gut microbiome spectrum by providing substrates that differentially promote the growth and activities of specific microbial communities [5–9]. Diet-microbiome interactions are consistently reproducible in clinical and animal studies [5, 8, 10, 11]. Specifically, many microbiome studies have focused on the effects of high-fat and/or high-sugar vs. low-fat and/or low-sugar diets on the gut microbial populations, particularly with respect to risk of chronic diseases and disorders such as obesity, type 2 diabetes, cardiovascular disease, and psychiatric disorders [12–15].

It is difficult to study long-term diet effects in human beings, as such studies rely on self-reported dietary intake collected using food frequency questionnaires which are not comprehensive or standardized, and nutrient intakes are estimated from these self-reports utilizing food composition tables. Therefore, actual nutrient intake is unknown. Several animal models including mice, rats, guinea pigs, and zebra fish have been used for studying diet-microbiome interactions. However, considering the potential health impact of diet effects on gut microbiome, the use of animal models demonstrably useful for studies of obesity, type 2 diabetes, cardiovascular disease, and psychiatric disorders [16] would be most helpful. Several population studies have indicated a beneficial effect of consumption of a Mediterranean diet on chronic diseases, and some evidence suggests that gut microbiota-mediated production of metabolites influencing metabolic health are involved. However, effects of Mediterranean diets have not been studied in animal models.

Cynomolgus monkeys (*Macaca fascicularis*) are widely used as models of diet-induced obesity, type 2 diabetes, coronary artery disease, and mood disorders [17–19]. Here we report the gut microbiome composition in healthy adult NHPs after long-term (2.5 years) consumption of Western- vs. Mediterranean-type diets.

## Materials and methods

### Subjects

The current work was conducted in socially housed adult female cynomolgus macaques maintained at the Wake Forest University Primate Center (established 1956; http://www.wakehealth.edu/ccpr). All animals were in apparently good health, free from gastrointestinal infections. The monkeys were randomized to one of two diet groups: Western or Mediterranean diet. Earlier studies of microbiome [20–23] used 3–15 animals to achieve >80% power and an α of 0.05, hence we selected *n* = 20 animals (*n* = 10 in each group), and no subjects were excluded from analysis. The monkeys were fed the experimental diets for 30 months (2.5 years), and water was accessible *ad libitum*.

### Diet

The western diet (hereafter, WD) consisted of lard, beef tallow, butter, egg, cholesterol, casein, lactalbumin, dextrin, high-fructose corn syrup, and sucrose; while the Mediterranean diet (hereafter, MD) comprised fish oil, olive oil, fish meal, butter, egg, black and garbanzo bean flour, wheat flour, V-8 juice, fruit puree, and sucrose. The detailed composition in terms of the percent of calories in these diets is provided in Table 1.

**Table 1:** Dietary composition of Western- and Mediterranean-style diets fed to the primates enrolled in this study.

	**Western diet^a^**	**Mediterranean diet^b^**
	**% of calories**	
Protein	16	16
Carbohydrate	54	52
Fat	31	32
	**% of total fats**	
Saturated	39	25
Monounsaturated	35	50
Polyunsaturated	25	25
ω6:ω3 fatty acids	15:1	3:1
Cholesterol (mg/Cal)	0.16^*^	0.15^*^
Fiber (% of diet)	9	13
Salt (g/100g diet)	0.75	0.15
	**Major ingredients differences**	
Ingredients	Lard	Fish oil
	Beef tallow	Olive oil
	Butter	Butter
	Egg	Egg
	Cholesterol	Fish meal
	Casein	Black and garbanzo bean flour
	Lactalbumin	Wheat flour
	Dextrin	V-8 juice
	High-fructose corn syrup	Fruit puree
	Sucrose	Sucrose

a *What we eat: Women 40–49, 2010-11; NHANES data published by USDA*.

b *[24]*.

* *About 256 mg/day*.

### Feeding

Individual feeding cages were fabricated and placed inside social group pens. The monkeys were taught to run into their individual feeding cages on voice command, were given 2 h to consume their diet, and released back into the social group pen. The monkeys consumed most of the diet in the first 30 min. Each monkey was offered 100 Cal/kg of diet.

### Sample collection

The NHPs were euthanized after the 30-month intervention period. Samples of rectal/anal contents of 10 randomly chosen monkeys from each of the diet groups (*n* = 20 total) were collected at necropsy and immediately placed in sterile tubes under aseptic conditions and stored at −80°C until further processing. All protocols related to the sampling, care and management of animals were reviewed and approved by the Institutional Animal Care and Use Committee at the Wake Forest School of Medicine.

### Microbiome analysis

Nearly 200 mg (wet weight) of each sample was used to extract genomic DNA by using the Qiagen DNA Stool Mini Kit (Qiagen, CA, USA) per manufacturer instructions. The bacterial 16S rRNA gene was amplified using the primers 515F (barcoded) and 806R, which flanked the V4 hypervariable region of bacterial 16S rRNA, in accordance with the Earth Microbiome Project protocol [25, 26] with the following minor modification. The PCR reaction consisted 25 μl of SYBR® Premix ExTaqTMII (Takara Bio, Shiga, Japan), 1 μl of each of the primers, 5 μl of DNA template, and 18 μl of RNase-free water (Invitrogen, Eugene, OR, USA). PCR conditions comprised an initial step at 50°C for 2 min and at 95°C for 10 min, with subsequent amplification steps at 95°C for 30 s, at 55°C for 30 s, and at 72°C for 90 s for repeated cycles on an Applied Biosystems® 7500 Real Time PCR System (Applied Biosystems). The amplification step was stopped before the fluorescent intensity reached a plateau. The resulting amplicons were purified using Agencourt® AMPure® XP (Beckman Coulter), quantified using the Qubit-3 fluorimeter (InVitrogen), normalized to an equal concentration (4 nm) and pooled together for 16S Miseq analysis. The sample pool was denatured, diluted to 8 pM and sequenced on an Illumina MiSeq platform, using a MiSeq Reagent Kit v3 (Illumina, San Diego, CA, USA) as described previously [27]. Sequencing procedure was monitored on the Illumina BaseSpace® website wherein analysis of the data generated on the Miseq platform was executed using the BaseSpace® 16S Metagenomics App (Illumina). Operational taxonomic unit (OTU) assignment to the Greengenes database was performed using the Quantitative Insights Into Microbial Ecology (QIIME) pipeline software package that enables microbial community analysis [26]. Demultiplexed R1 and R2 sequencing read files were obtained from the Illumina BaseSpace® website and the Illumina reads were quality-filtered, clustered and analyzed using default parameters in QIIME. A total of 1.33 million reads (mean = 51665.50; standard deviation = 21791.5) were generated after filtering. Paired-end reads were joined together with fastq_join_paired_ends.py and split_libraries_fastq.py scripts. The assembled sequences were grouped into OTUs at a sequence similarity of 97% identity and sequences were classified into the taxonomical levels based on the Greengenes 16S rRNA gene database (http://greengenes.secondgenome.com). Representative OTU sequences were aligned to a Greengenes reference alignment [25]. *De novo* OTUs were classified using RDP classifier and the Greengenes reference set with a minimum 80% confidence threshold [28]. Samples were rarified at an even sequencing depth of 10,000 reads per sample for subsequent downstream analyses.

### Data analysis

Taxonomy assignment and diversity analyses were computed through QIIME with default settings to compare bacterial species richness between the two diet groups. Bacterial composition of each sample was measured at various taxonomic levels using QIIME. Alpha diversity (rarefaction curve for observed OTUs, Chao1, PD_Whole_Tree and Shannon) indices were computed with core_diversity_analysis.py script. Beta diversity was generated within QIIME by using weighted and unweighted Unifrac distance matrices. UniFrac distances are appraised as the distance between bacterial communities explaining phylogenetic relationship between bacteria. Principal components analysis (PCA), an unsupervised analysis that allows estimation and visualization of sample distribution based on UniFrac distance patterns, was performed to determine the influence of dietary treatments on the overall microbiome composition of the samples. PCA plots were visualized using EMPeror version 0.9.3-dev. Unweighted PCA was used to determine if the NHP microbiome phenotypes clustered by the type of diet. The data of bacterial diversity and abundance between the two diet groups were compared by using non-parametric analyses in R statistical software package (version 3.4.3; https://www.r-project.org/). Statistically significant differences between two groups were calculated by Mann–Whitney *U-*test with Bonferroni correction. Statistical significance in alpha-diversity was calculated by non-parametric two-sample *t*-tests with 9999 Monte-Carlo permutations. Difference in beta-diversity was examined by permutational multivariate analysis of variance (PERMANOVA), a permutation-based extension of multivariate analysis of variance to a matrix of pairwise distance that partitions the inter-group and intra-group distances. Hierarchical clustering maps based on average linkage on Euclidean distance were constructed in R using “ggplots” library and ward.D2 method. Results are expressed as mean ± SEM. Unless otherwise stated, a value of *P* < 0.05 was considered statistically significant.

## Results

**Figure 1:**
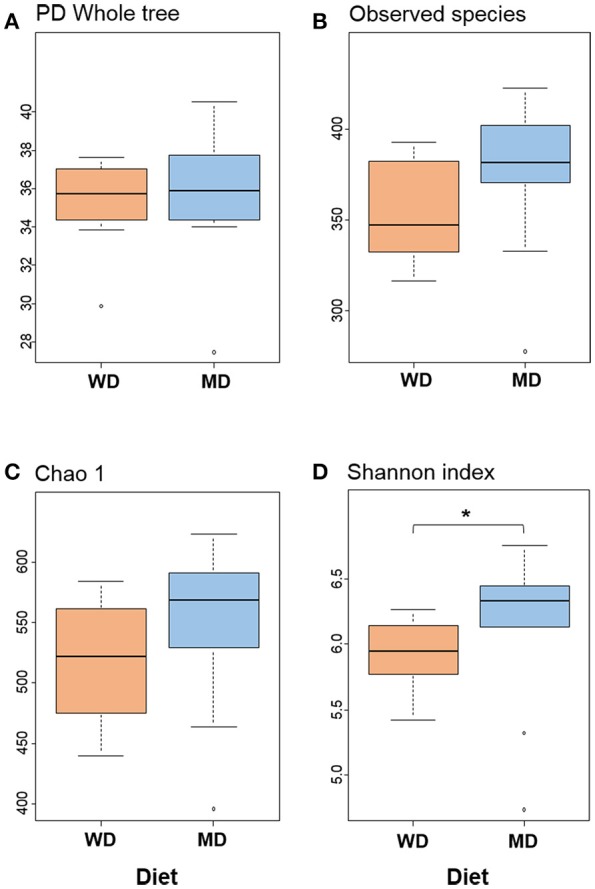
Bacterial diversity indices **(A–D)** in the distal gut samples of non-human primates consuming either a Western-style diet (WD; *n* = 10) or a Mediterranean-style diet (MD; *n* = 10) for a period of 30 months. ^*^P < 0.05; Mann–Whitney *U*-test (Monte Carlo permutation).

Diversity metrics viz. Shannon, Chao1, observed OTU, and phylogenetic diversity indices demonstrated varying but similar trend for higher microbiome diversity in MD vs. WD consumers (Figure 1; Supplementary Figure S1). Chao1 index, observed species and phylogenetic diversity were insignificantly higher in MD group compared with WD group; while the Shannon Diversity index, which constitutes both microbial richness and abundance, was found to be significantly (*P* < 0.05) higher in MD vs. WD consumers (Figure 1).

**Figure 2:**
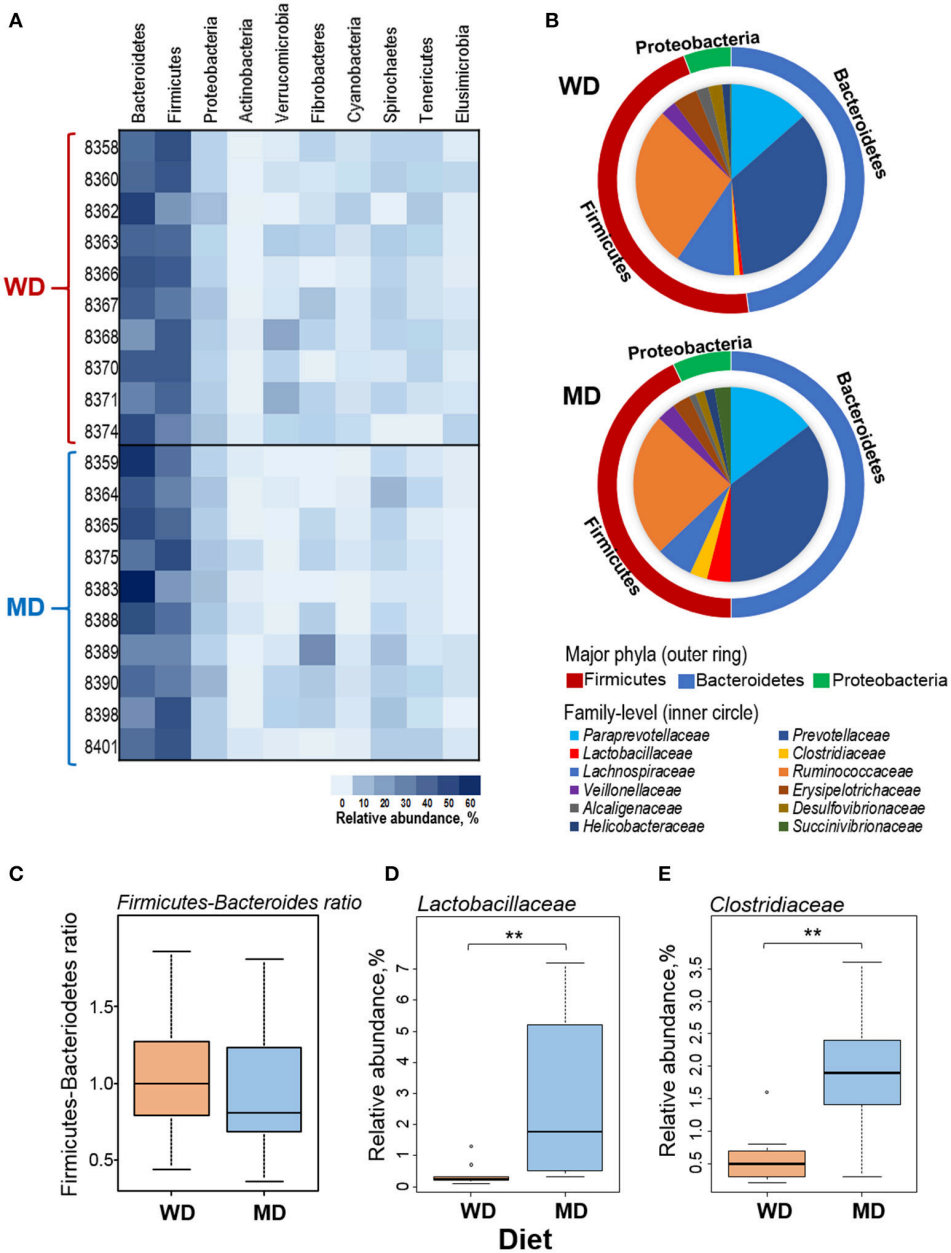
**(A)** Heat-map depicting the relative abundance of major phyla observed in 20 primates consuming either a Western-style diet (WD; *n* = 10) or a Mediterranean-style diet (MD; *n* = 10) for a period of 30 months. **(B)** Pie-charts showing the comparison in the relative abundance of top three phyla and major families detected within these phyla. **(C–E)** Box-plots showing the comparison in the *Firmicutes* to *Bacteroidetes* ratio **(C)** and the relative abundance of families *Lactobacillaceae* **(D)** and *Clostridiaceae* **(E)** between WD and MD groups. ^**^P < 0.001; Mann–Whitney *U*-test (Monte Carlo permutation).

Overall, at phyla level, the microbiome of these NHP was predominated by Bacteroidetes (42%), Firmicutes (38%), and Proteobacteria (5%), followed by Verrucomicrobia, Fibrobacteres, Actinobacteria, Cyanobacteria, Spirochaetes, Tenericutes, and Elusibacteria (Figure 2A). Compared with WD group, MD group demonstrated slightly higher abundance of Bacteroides (44 vs. 41%), Proteobacetria (6.2 vs. 4.9%), Fibrobacteres (4.5 vs. 2.4%), and Spirochaetes (4.6 vs. 2.3%) and lower abundance of Firmicutes (37 vs. 40%) and Verrucomicrobia (0.7 vs. 4.6%) (Figures 2A,B). Accordingly, the Firmicutes to Bacteroides ratio was found to be relatively lower in MD group (0.86) compared to that in WD group (0.97) (Figure 2C). Within the three major phyla (i.e., Bacteroidetes, Firmicutes, and Proteobacteria), *Paraprevotellaceae, Prevotellaceae, Lactobacillaceae, Clostridiaceae, Lachnospiraceae, Ruminococcaceae, Veillonellaceae, Erysipelotrichaceae, Alcaligenaceae, Desulfovibrionaceae, Helicobacteraceae*, and *Succinivibrionaceae* represented the top 12 most abundant families (Figure 2B). Among these, *Clostridiaceae* and *Lactobacillaceae* were found to be significantly more abundant in MD vs. WD group (Figures 2D,E). The details of other major taxa detected at the level of phyla, class and order are provided in Supplementary Figure S2.

**Figure 3:**
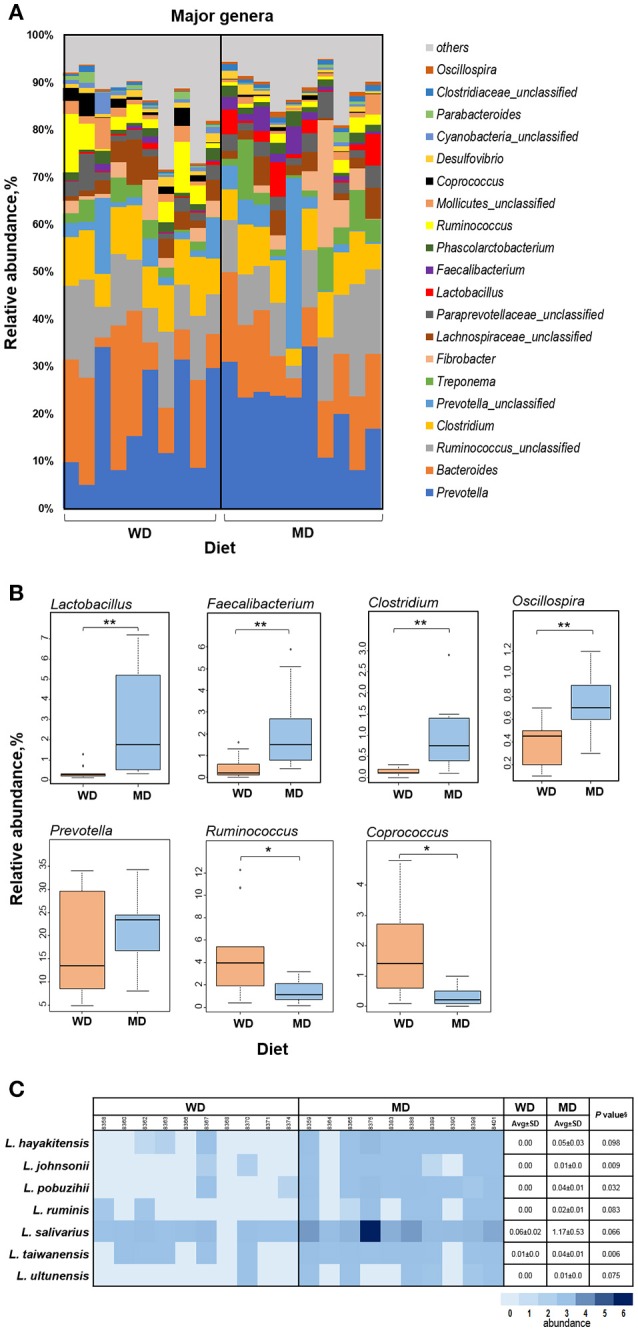
**(A)** Bar graph showing the relative abundance of major genera observed in the gut microbiome of 20 primates consuming either a Western-style diet (WD; *n* = 10) or a Mediterranean-style diet (MD; *n* = 10) for a period of 30 months. **(B)** Box-plots showing the comparison of relative abundance of major genera that showed a notable difference between WD and MD groups. ^*^P < 0.05; ^**^P < 0.005; Mann–Whitney *U*-test (Monte Carlo permutation). **(C)** Relative abundance of major species belonging to the genus *Lactobacillus* in WD and MD groups. ^§^Unpaired *t*-test.

Figure 3A shows the relative abundance of top 20 most abundant genera observed in this cohort of NHPs. The overall spectrum of these genera appeared to be slightly different between the two groups wherein several genera showed differences between the two diet groups. Among these, *Lactobacillus, Faecalibacterium, Clostridium, Oscillospira*, and *Prevotella* exhibited the most prominent difference with significantly or insignificantly higher abundance in MD vs. WD group (Figure 3B) whereas *Ruminococcus* and *Coprococcus* exhibited an opposite trend, i.e., a significantly lower relative abundance in MD vs. WD group (Figure 3B). However, PCA analysis did not show any considerable clustering of the gut bacterial communities between the two diet groups (Supplementary Figure S4).

The two diet groups also exhibited differences in the abundance of several species belonging to these genera (Supplementary Table S1), among which *Lactobacillus* species demonstrated the most prominent difference (Figure 3C). Although *L. salivarius* represented the most abundant *Lactobacillus* species, the abundance of all of the *Lactobacillus* species detected was higher in MD compared to WD group (Figure 3C).

## Discussion

The gut of NHPs is inhabited by a complex and dynamic bacterial community and the compositional as well as functional array of this community can be influenced by various intrinsic and extrinsic elements such as host diet, physiology, geography, and clinical health [29]. The gut microbiome of NHPs has been found to be more similar to those of human primates than to other animals [30]. For instance, the human gut is inhabited by microorganisms belonging to nine different divisions of Bacteria: Firmicutes and Bacteroides (the predominant and most abundant), Actinobacteria, Fusobacteria, Proteobacteria, Verrucomicrobia, Cyanobacteria, Spirochaetes and VadinBE97 [31, 32]. In the present study, we identified Bacteroidetes, Firmicutes, (the most abundant), Proteobacteria, Actinobacteria, Verrucomicrobia, Fibrobacteres, Cyanobacteria, Spirochaetes, and Tenericutes as the top nine bacterial phyla in these NHPs. Hence, investigation of microbes dwelling in the gastrointestinal tract of these NHPs might provide important clues about the characteristics of these bacterial groups in the human gut. It is also likely that the microbiome composition of these captive monkeys is more humanized than that of wild free-living NHPs consuming more native diets due to the human-like diets they were fed [33].

Western- and Mediterranean-type diets are generally studied and exemplified as unhealthy and healthy dietary habits, respectively. Mounting evidence shows that diets rich in fiber and unsaturated fatty acids are important for maintenance of a diverse and healthy gut microbiome. Although we did not observe a remarkable difference in the overall microbiome signature between the two groups; we noted several interesting differences at family, genus, and species levels. Consistent with previous findings in different animal species including humans [3, 4, 6, 34], this demonstrates that diet can have a strong influence on the gut microbiome communities in NHPs and supports the notion that diet can affect the gut microbiota composition without causing dramatic changes in the overall microbial diversity [30, 34]. The higher Shannon diversity index in MD vs. WD group is interesting and could be ascribed to higher proportion of fiber in MD [3, 33, 35, 36]. The responses of NHP gut microbiome toward these diets might differ from those of human microbiome [37]; although we found several patterns of diet-induced differences similar to those reported previously in humans or small animals. This may be exemplified by higher abundance of *Oscillospira* (a genus belonging to the *Ruminococcaceae* family) in MD vs. WD group. *Oscillospira* species are generally prevalent in the gut of ruminants consuming diets rich in complex plant polysaccharides and are considered to be bacteria adapted to subsisting on vegetable-rich diet such as the MD [38]. Interestingly, a higher intestinal carriage of *Oscillospira* has also been reported in humans consuming a MD [39].

The relatively lower Firmicutes-Bacteroides ratio and higher abundance of genera *Clostridium* and *Prevotella* might be expected, in agreement with previous studies reporting that consumption of MD is associated with positive alterations in the gut microbiota composition with increased abundance of *Bacteroides, Clostridium*, and *Prevotella* [6, 40, 41]. Particularly, a lower Firmicutes-Bacteroides ratio can also be ascribed to high fiber proportion in MD. Hierarchical clustering also generated two clusters of *Firmicutes* and *Bacteroides* abundance wherein the *Firmicutes*-rich cluster had higher proportion of WD subjects (64%; 7 out of 11) while *Bacteroides*-type cluster was dominated by MD subjects (67%; 6 out of 9) (Chi-square, *P* = 0.17) (Supplementary Figure S3). Interestingly, high prevalence of Bacteroidetes and reduced abundance of Firmicutes has also been demonstrated in humans and other mammals and are linked to higher fiber intake and less intake of high-glycemic index sugars [6, 42, 43]. Species of *Bacteroides* and *Prevotella*, the major constituents of gut microbiota, have been linked with high-polysaccharide diets [33, 43], whereas WD is linked with increased *Firmicutes* and reduced *Bacteroides* population [6]. The higher abundance of *Faecalibacterium* genus (Figure 3B) as well as *Faecalibacterium prausnitzii* (Supplementary Table S1) also coincides with previous human studies reporting increased abundance of several butyrate-producing bacteria including *Faecalibacterium* in human subjects consuming a Mediterranean-style diet [39, 44]. Interestingly, the abundance of *F. prausnitzii* is found to be decreased and negatively correlated with inflammatory markers in type-2 diabetes patients [39, 45, 46]; whereas MD has been found to increased in abundance in patients with metabolic syndrome[39]. This indicates that the beneficial effects of specific Mediterranean-style diets on host health might be conveyed at least partly via amelioration of gut dysbiosis. This, in turn, might also suggest a preventative effect of MD against type-2 diabetes and should be an interesting topic for prospective studies to further investigate diet-induced alterations in gut microbiome and metabolome in particular context to the prevention/ amelioration of metabolic disorders.

Generally, Mediterranean-style diets favor the intestinal population of lactic acid bacteria through higher proportion of fermented foods (e.g., yogurt) in these diets. Although the diets used in the present study did not include any fermented ingredient *per se*, we still observed significantly higher abundance of genus *Lactobacillus* in MD group. Diets containing complex carbohydrates, e.g., prebiotics, have been shown to favor the proliferation of beneficial bacteria including *Lactobacillus* sp. [47, 48] and *Faecalibacterium* sp. [49] in the gut, as reported in the present study. Furthermore, omega-3 fatty acids, which are present in higher proportion in MD, are also known to promote the population of several beneficial bacterial groups including lactobacilli that populate the distal gut, a site for the metabolism of mono- and poly-unsaturated fatty acids [50–52]. On the other hand, western-style high-fat diets have been shown to reduce the gut *Lactobacillus* population [12, 53, 54].

Despite being present in subdominant abundance; lactobacilli represent an important bacterial group within the human gut bacterial community. Ecological studies have validated their occurrence in the gastrointestinal tract of a wide variety of animals including mammals, rodents, birds, ungulates, lagomorphs, and insects. However, data on the composition of *Lactobacillus* community in NHPs is lacking. Lactobacilli are highly prevalent in food and dairy products, although primitive sources might have been fecal contamination and/or deliberate probiotic addition [55]. For that reason, *Lactobacillus* species are generally considered as host species-specific and could also be categorized as human- or non-human type [56]. In these contexts, the investigation of *Lactobacillus* community in the gut of NHPs could be an interesting area for further studies, particularly owing to their long and close evolutionary connection with the human gastrointestinal tract [57]. In addition, such exploration might also pave way for discovery of novel strains that could also be exploited as probiotics for NHPs. Nevertheless, more inclusive and broader studies are required to corroborate this relationship as well as to identify the core *Lactobacillus* taxa for specific NHP species and to validate whether and how these taxa are related to the host health, nutrition and disease. Based on our data, *L. salivarius* appears to be the major *Lactobacillus* group in these monkeys; however further investigation by both culture-dependent and -independent methods is needed to establish this finding as well as to examine whether and how *Lactobacillus* flora varies during different stages of NHP's lifespan as it does in human hosts [9, 58].

Mediterranean- and Western-type diets are well known to promote different metabolic phenotypes in several animal and human studies, wherein perturbations in the gut microbiome induced by western-style diets have been shown to play causal role in several gut-related diseases including adiposity, type 2 diabetes, and other metabolic syndromes [3, 12]. MD and WD subjects in the present study differed in food consumption, insulin sensitivity, BMI and several physiological markers between the two groups (data not shown here). Future studies (in-progress) will more closely evaluate relationships between the microbiome characteristics and an array of metabolic phenotypes along with metabolomic signature being evaluated in the parent study.

The strengths of the present study are; (a) the intervention period (2.5 years) is controlled and relatively much longer compared to most other dietary interventions in either animal model or randomized human trials, (b) relative to rodent diets, the formulation of diets given to NHPs is quite similar in composition to that of typical human diet, and (c) the method of fecal sample collection, which involved near sterile collection of rectal/colonic contents at the time of necropsy, provided a fresh specimen representing the distal gut microbiome while evading the possibility of contamination from skin or environmental flora as might occur with opportunistic fecal collections following defecation. However, the study also had some limitations, including the lack of baseline data which would allow assessments of microbiome changes occurring due to the transition from a chow diet to more human like diets. Given that the animals were randomized before enrollment to the two different diets, chances of any potential bias in results are minimal. The number of subjects (*n* = 20) is modest compared to large epidemiological studies, however well controlled and reasonable with respect to many rodent studies where the number typically ranges from 5 to <10.

In summary, the differences observed here in the gut microbiome of NHPs consuming either a Western or Mediterranean diet suggest that this model will be useful in further studies aimed at understanding the diet-microbiome-health interactions in primates.

## Author contributions

CS, TR, HY: conceived and designed study; RN: performed microbiome experiments; RN, HY: analyzed data; RN, HY: interpreted data; CS, SA, TR, KM: coordinated animal studies and sample collections; RN, HY, CS, TR: wrote and revised manuscript; RN, CS, SA, TR, KM, MV, HY: approved final version of manuscript.

## Conflict of interest statement

The authors declare that there are no competing interests, either financial or otherwise, with regard to this manuscript.
