# Supplementary material for: Gut Microbiome Composition in Non-human Primates Consuming a Western or Mediterranean Diet
Source: Front Nutr. 2018 Apr 25;5:28. doi: 10.3389/fnut.2018.00028 (PMC5996930; doi:10.3389/fnut.2018.00028)
Supplement: Supplementary file 1 [file Presentation_1.pdf]

Supplementary Figures

Nagpal et al.

SUPPL. FIGURE S1. Gut bacterial diversity metrics (a-d) as a function of sequence depth in primates consuming consuming either a Western-style diet (WD; n=10) or a Mediterranean-style diet (MD; n=10) for a period of 30 months. Values are represented as Mean ± SEM.

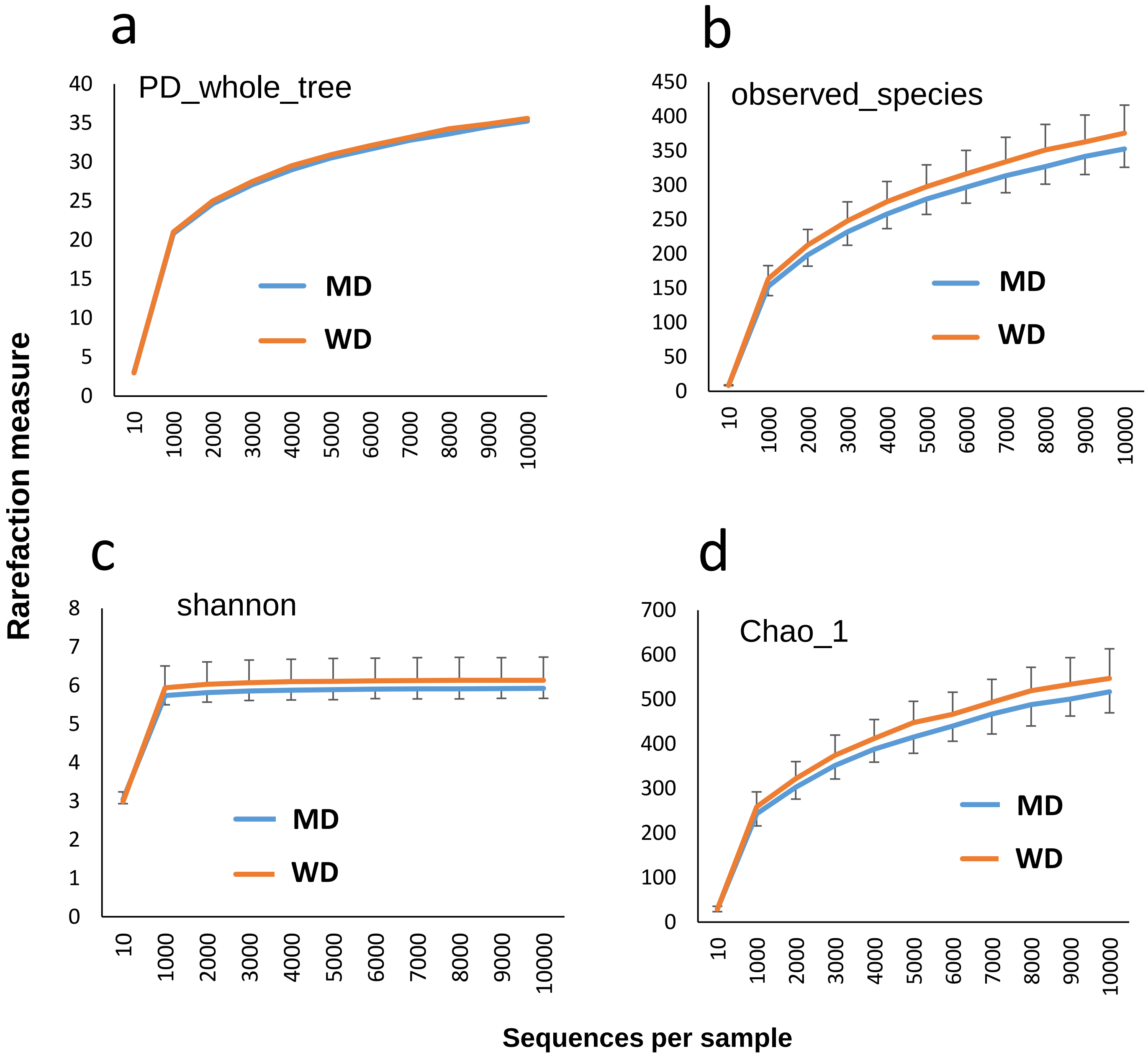

SUPPL. FIGURE S2. Bar graph showing the relative abundance of major bacterial phyla (a), classes (b) and orders (c) observed in the gut microbiome of 20 primates consuming either a Western-style diet (WD; n=10) or a Mediterranean-style diet (MD; n=10) for a period of 30 months.

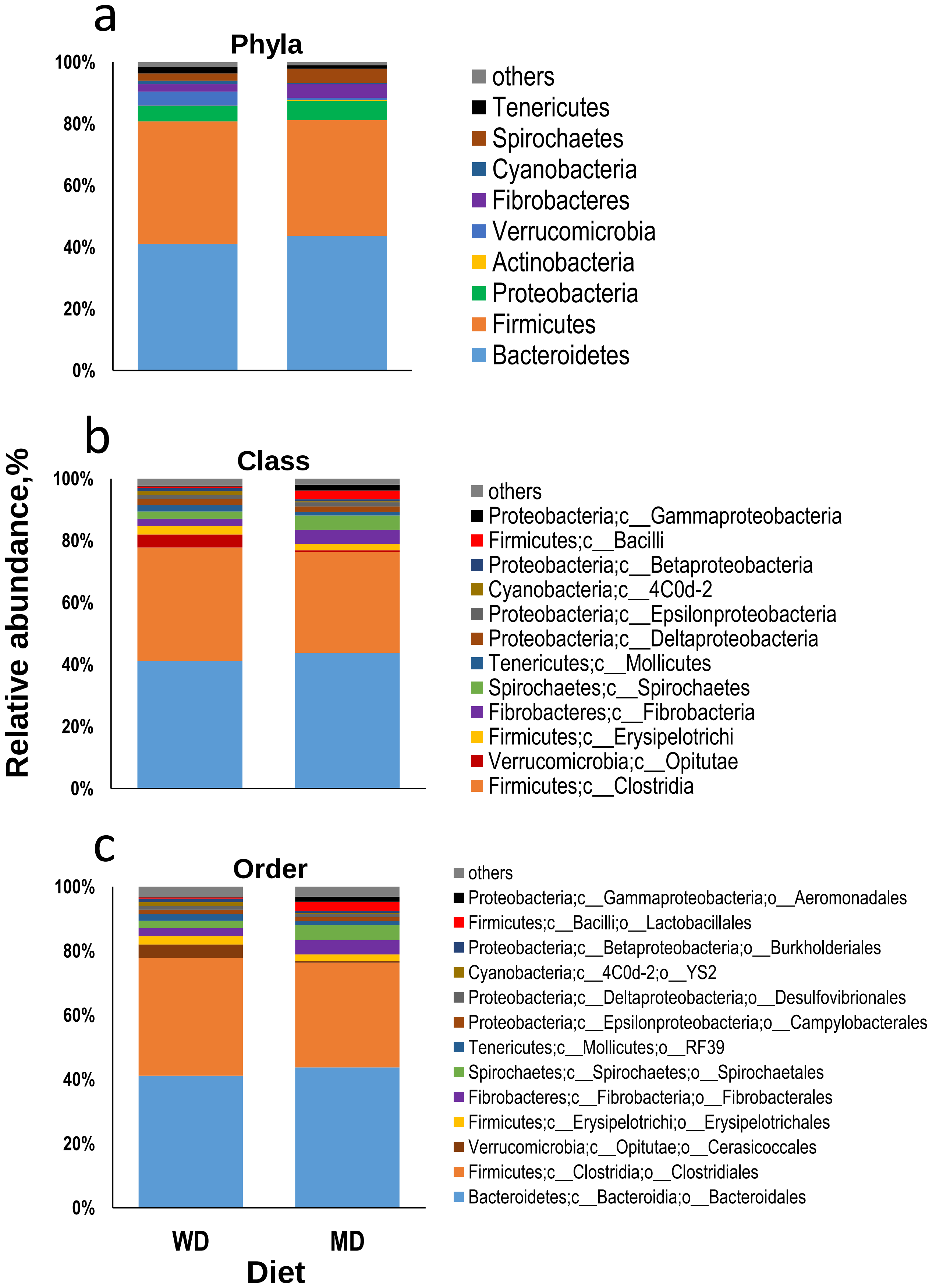

SUPPL. FIGURE S3. Heat-map depicting the clustering based on the relative abundance of major bacterial phyla observed in the gut microbiome of 20 primates consuming either a Western-style diet (WD; n=10) or a Mediterranean-style diet (MD; n=10) for a period of 30 months.

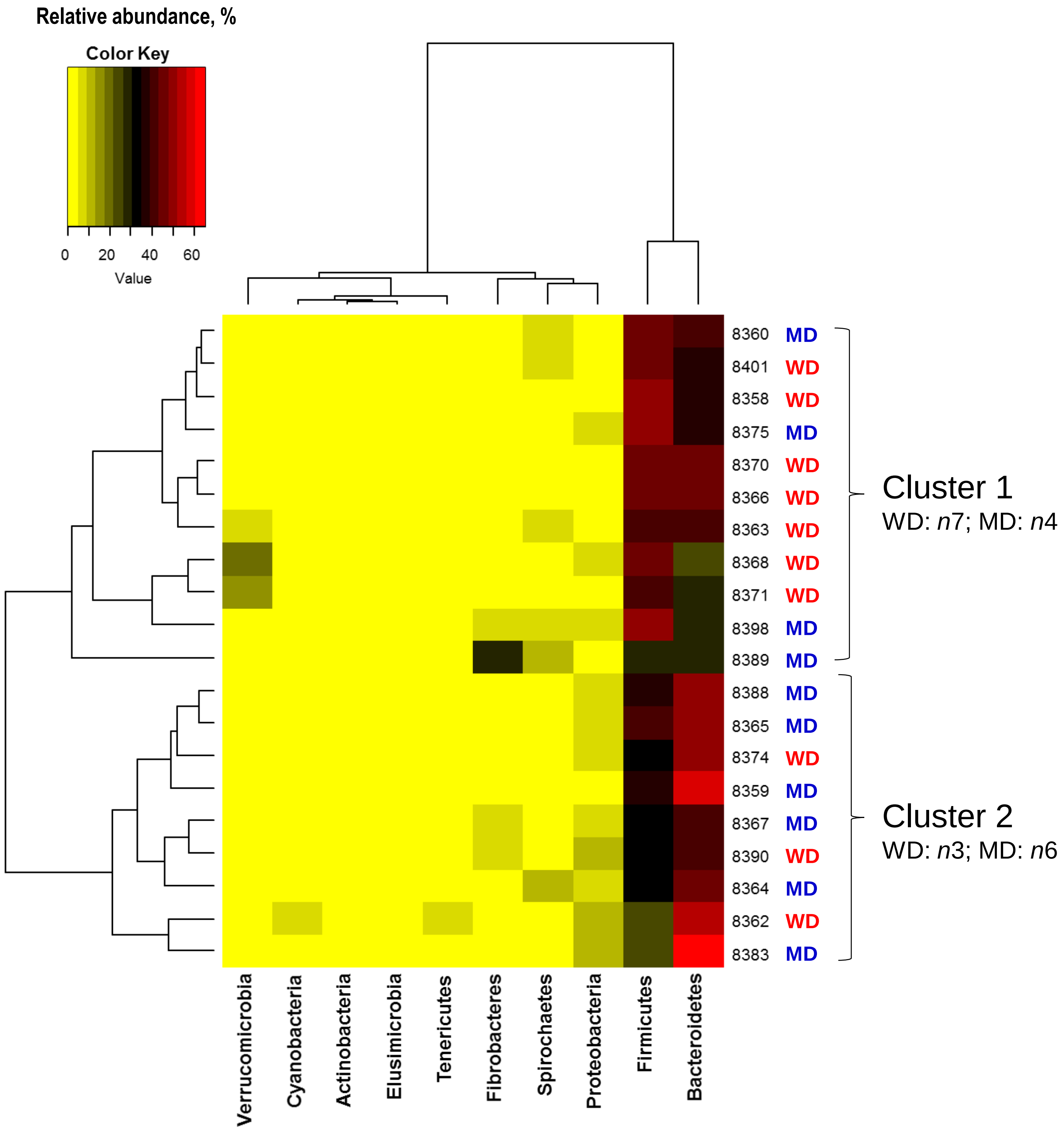

SUPPL. FIGURE S4. Principal coordinate analysis (PCA) of bacterial communities in the gut microbiome of 20 primates consuming either a Western-style diet (WD; n=10) or a Mediterranean-style diet (MD; n=10) for a period of 30 months.

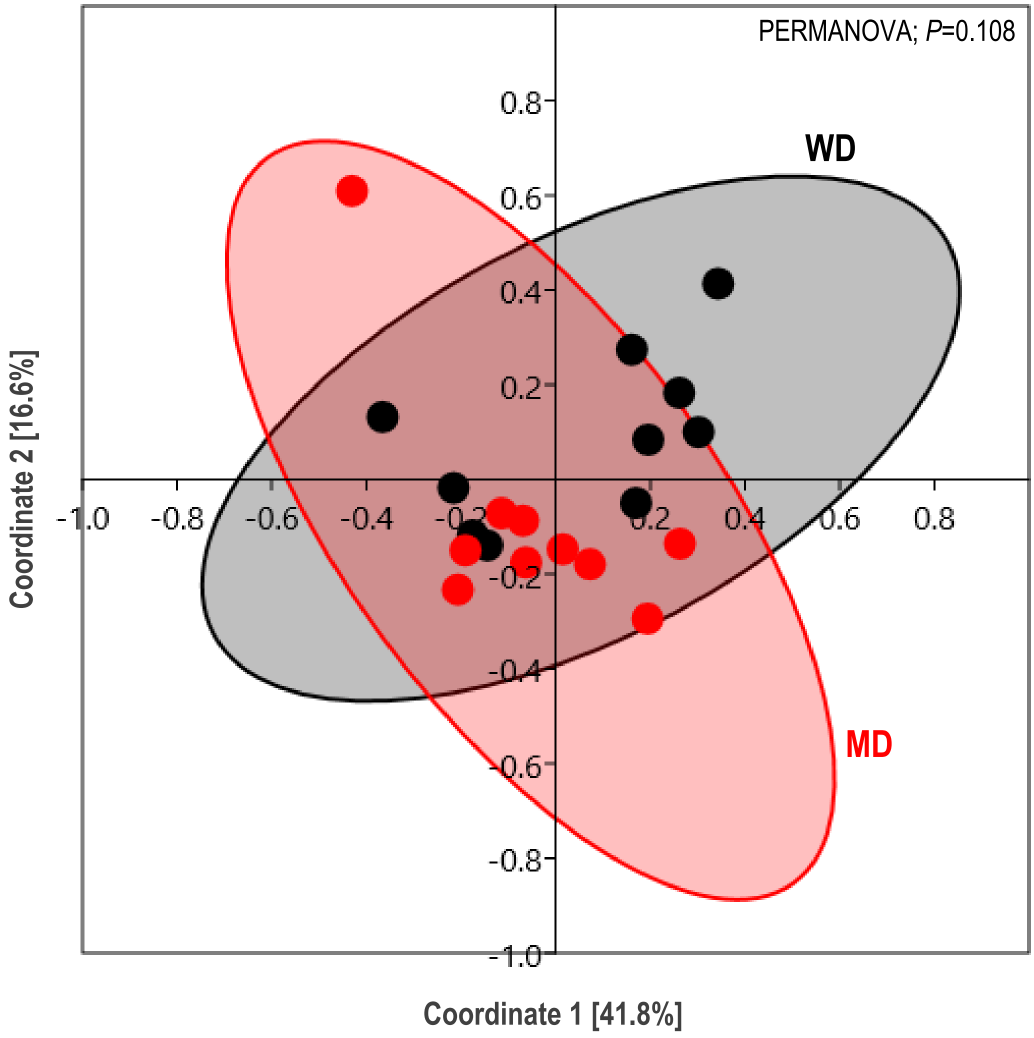

Table S1. Differences in the relative abundance (%) of bacterial species in the gut microbiome of primates consuming a Western-diet (WD) or a Mediterranean diet (MD) for a period of 30 months. Only species exhibiting noticeable difference between the two groups are shown here. *Unpaired *t*-test.

	WD		MD		*P* value*
	Avg.	SEM	Avg.	SEM	
Bacteroides salanitronis	0.00	0.00	0.06	0.02	0.017
Bacteroides xylanisolvens	0.16	0.04	0.32	0.07	0.047
Clostridium alkalicellulosi	1.21	0.25	0.49	0.13	0.018
Coprococcus eutactus	0.01	0.01	0.42	0.21	0.084
Erysipelothrix muris	3.61	0.86	0.94	0.20	0.012
Faecalibacterium prausnitzii	0.65	0.23	2.92	0.83	0.024
Lachnospira pectinoschiza	0.07	0.01	0.22	0.08	0.080
Prevotella multisaccharivorax	0.02	0.00	0.41	0.17	0.049
Prevotella shahii	0.04	0.02	0.12	0.05	0.099
Rhodothermus clarus	1.30	0.14	0.61	0.14	0.003
